# Development and Validation of a Short Version Eye‐Tracking Paradigm for the Screening and Diagnosis of Autism Spectrum Disorder in Qatar

**DOI:** 10.1002/aur.70242

**Published:** 2026-03-30

**Authors:** Fouad Al Shaban, Iman Ghazal, Fatema Al‐Faraj, Sarah Aqel, I. Richard Thompson

**Affiliations:** ^1^ Neurological Disorders Research Center Qatar Biomedical Research Institute, Hamad Bin Khalifa University, Qatar Foundation Doha Qatar; ^2^ College of Health and Life Sciences, Hamad Bin Khalifa University, Qatar Foundation Doha Qatar; ^3^ The North East and Yorkshire NHS Genomic Laboratory Hub Leeds UK

**Keywords:** autism spectrum disorder, diagnosis, eye‐tracking, gaze behavior, rapid screening

## Abstract

Objective behavioral assessments for autism spectrum disorder (ASD) are often time‐intensive and require substantial clinical expertise. Eye‐tracking–based paradigms offer quantifiable measures of social attention that can complement traditional tools. The current study builds on our previously validated Arabic‐language Autism Index (AI) by developing and validating a 4 min short version designed to improve feasibility in clinical and community settings while maintaining diagnostic accuracy. A total of 236 participants (127 with ASD, 109 non‐autistic controls including those with developmental delays (DD)) aged 1–16 years were assessed using an eye‐tracking paradigm consisting of 19 short dynamic videos depicting social and non‐social scenes. The AI was computed as the ratio of dwell time toward social versus non‐social stimuli. Diagnostic classification was established using ADOS‐2 and SCQ. Reliability and validity were assessed using Cronbach's *α*, Pearson's *r*, and ROC analyses, including age‐stratified performance and comparison with the original 10 min version. Feasibility was assessed by the proportion of valid stimuli. The short‐version AI demonstrated excellent internal consistency (*α* = 0.91) and test–retest reliability (*r* = 0.83). Diagnostic accuracy was high (AUC = 0.878, SE = 0.023), with age‐stratified AUCs ranging from 0.846 to 0.939. AI scores correlated strongly with ADOS‐2 severity (*r* = 0.54, *p* < 0.001) and SCQ total scores (*r* = 0.43, *p* < 0.001). Compared with the 10 min original version (AUC = 0.73), the short paradigm achieved higher accuracy and feasibility (valid stimuli: 89% vs. 80%). The current eye‐tracking paradigm demonstrates promising diagnostic performance while substantially reducing assessment time and cognitive demand. The findings provide initial evidence supporting its potential as a scalable and cross‐cultural tool for ASD screening and diagnosis, with further validation in independent and clinical cohorts supporting its translation into routine clinical practice.

## Lay Summary

Children with autism often show noticeably different patterns of looking at people and objects compared to other children. Eye‐tracking technology can measure these differences and help clinicians identify autism in a more objective way. In this study, we tested a 4 min version of an eye‐tracking test that was designed to be easier for children to complete. Our findings show that this much faster test remains reliable and useful for autism screening and diagnosis, especially in clinics and schools where time and attention are limited.

**Abbreviations**
ADOS‐2: Autism Diagnostic Observation Schedule, Second Edition
AI: Autism Index
ASD: autism spectrum disorder
AUC: area under the curve
DSM‐5: Diagnostic And Statistical Manual of Mental Disorders, Fifth Edition
ROC: receiver operating characteristic
ROI: region of interest
SCQ: Social Communication Questionnaire

## 1 Introduction

Autism spectrum disorder (ASD) is a complex neurodevelopmental condition marked by challenges in social communication and interactions, with the presence of repetitive behaviors and interests (Lord et al. 2018). Early identification of ASD is critical, as timely intervention significantly improves long‐term developmental outcomes (Hyman et al. 2020). Despite advances in clinical assessment, current diagnostic methods—such as the Autism Diagnostic Observation Schedule (ADOS‐2) and Autism Diagnostic Interview–Revised (ADI‐R)—remain dependent on specialized training and subjective interpretation, limiting scalability and contributing to delays in diagnosis, particularly in culturally diverse and resource‐limited contexts (Huda et al. 2024). Current diagnostic approaches lack an objective marker capable of delivering immediate interval‐scale measurements with consistently high reliability across the diverse spectrum of behaviors exhibited by individuals with ASD. Consequently, the creation of objective assessment tools holds significant promise for advancing clinical evaluations by complementing and enhancing the existing diagnostic methods (Al‐Shaban et al. 2023). As a result, research efforts have increasingly focused on identifying quantifiable indicators to improve the accuracy and reliability of ASD diagnostics (Ansel et al. 2019). Among these, emerging evidence suggests that incorporating technologies like eye‐tracking can significantly refine the diagnostic process by providing quantifiable, unbiased measures to support existing assessment methods (Frazier et al. 2016).

Eye‐tracking offers an objective, quantifiable approach to measuring social attention—a foundational process that influences language, learning, and social cognition (Frazier et al. 2021). Individuals with ASD typically exhibit atypical gaze behavior, characterized by reduced attention to social stimuli and increased focus on non‐social or geometric patterns (Frazier et al. 2017). Longitudinal and cross‐sectional studies have demonstrated that reduced spontaneous attention to faces and eyes reliably differentiates autistic from non‐autistic children and predicts later social symptom severity (Jones et al. 2008; Jones and Klin 2013). These attentional patterns emerge early in development and remain stable across age groups and cultures, suggesting that gaze behavior may serve as a robust neurobehavioral marker of ASD (Chita‐Tegmark 2016; Kaliukhovich et al. 2020). In addition, they align with early descriptions of autism (Kanner 1968) and are considered critical markers in all established diagnostic tools (Lord et al. 2012; Rapin 1997). Recent studies have demonstrated that children with ASD often exhibit a preference for geometric patterns over social images (Pierce et al. 2011, 2016). In addition, social stimuli analyzed through gaze tracking have shown potential to differentiate individuals with ASD or other DD from neurotypical controls (Chevallier et al. 2015; Guillon et al. 2014). This attentional bias can be quantified using eye‐tracking devices, providing measurable data that support early diagnosis (Keehn et al. 2024). Moreover, eye‐tracking facilitates the assessment of intervention outcomes by providing objective measures of changes in social attention over time. This dual functionality highlights the potential of eye‐tracking as both a diagnostic and screening tool in ASD research and clinical practice (Hamner and Vivanti 2019). Building on this foundation, several studies have demonstrated that eye‐tracking data can be aggregated into an Autism Index (AI)—a single metric capturing relative gaze preference for social versus non‐social information—with diagnostic accuracy comparable to conventional screening tools (Frazier et al. 2018; Al‐Shaban et al. 2023). Beyond ASD‐specific applications, growing evidence suggests that eye‐tracking–derived measures of social attention represent a broader, transdiagnostic neurodevelopmental risk marker. Frazier et al. demonstrated that reduced social visual engagement is consistently observed across neurodevelopmental conditions and cultural contexts, supporting its utility as an objective, scalable biomarker (Frazier et al. 2021). More recently, remote eye‐tracking approaches have been shown to reliably capture social attention patterns in both idiopathic neurodevelopmental disability and neurogenetic syndromes, highlighting the feasibility of deploying such measures beyond traditional laboratory settings (Frazier et al. 2025). In parallel, recent large‐scale studies have further advanced the use of eye‐tracking as an objective diagnostic aid for autism. In two independent investigations, eye‐tracking–based measures of social visual engagement demonstrated strong agreement with expert clinical diagnosis and robust diagnostic accuracy in early childhood populations (Jones, Klaiman, Richardson, Aoki, et al. 2023; Jones, Klaiman, Richardson, Lambha, et al. 2023). Collectively, this body of work highlights the growing consensus that eye‐tracking–derived measures of social attention can serve as scalable, objective complements to traditional clinician‐administered assessments.

In our previous work, we developed and validated an Arabic‐language eye‐tracking paradigm that culturally adapted the AI for use among Arabic‐speaking children (Al‐Shaban et al. 2023). That study demonstrated strong internal reliability (*α* = 0.91), high test–retest stability, and cross‐cultural validity in distinguishing ASD from non‐autistic controls (AUC = 0.73). However, feedback from clinicians and families revealed practical barriers to routine implementation—particularly the test's length (≈10 min) and the associated attentional demands for young children with ASD. These constraints limited feasibility in clinical workflows and large‐scale screening programs. To address these limitations, the present study developed and validated a short, 4 min version of the same eye‐tracking paradigm. The new version was designed to reduce assessment time and participant fatigue while preserving the diagnostic accuracy and psychometric properties of the original AI. The shortened paradigm retained the most diagnostically informative stimuli from the longer version and was optimized for use in both clinical and community‐based settings.

The study aimed to (a) evaluate the diagnostic performance, reliability, and concurrent validity of the short AI relative to clinical diagnostic standards (ADOS‐2, SCQ, and DSM‐5), and (b) evaluate whether comparable diagnostic performance could be achieved relative to the previously validated long version. We hypothesized that the short AI would (1) demonstrate an Area Under the Curve (AUC) ≥ 0.80 in differentiating ASD from non‐ASD participants, (2) maintain strong internal consistency and test–retest reliability, and (3) show significant positive correlations with established measures of ASD symptom severity. By confirming these hypotheses, this study aims to advance eye‐tracking from a research‐based measure to a clinically practical, time‐efficient diagnostic aid suitable for broader application in early ASD detection.

## 2 Methods

### 2.1 Participants

Participants in this study ranged between 1 and 16 years old and were divided into two primary groups: (1) children with a confirmed diagnosis of ASD (*n* = 127) and (2) non‐ASD controls (*n* = 109), which included both typically developing (TD) children and those with DD. The broad age range was intentionally selected to evaluate the robustness of the AI across developmental stages, as atypical social attention patterns associated with ASD emerge early and persist across development (Chawarska et al. 2013; Jones and Klin 2013).

ASD participants were recruited from local neurodevelopmental clinics and specialized centers in Qatar. All had a prior clinical diagnosis of ASD or were referred for evaluation based on suspected social or communication difficulties. Diagnostic status was verified using standardized assessments described below in Section 2.2. The control group were recruited from various sources, including siblings of children with ASD (*n* = 34), primary care clinics, and research networks. The control group included TD participants, defined as having no current or past developmental or psychiatric conditions, DD participants, defined as individuals with speech or cognitive delays and/or other neurodevelopmental or neuropsychiatric diagnoses (e.g., ADHD, selective mutism, sensory processing disorder), excluding ASD. Due to the relatively small number of children in the DD group (*n* = 16), these participants were combined with the neurotypical group for comparison. Inclusion of children with DD was consistent with prior validation studies of the AI and aimed to assess specificity against non‐ASD neurodevelopmental conditions (Al‐Shaban et al. 2023). Sensitivity analyses excluding the DD subgroup yielded comparable diagnostic performance, confirming that their inclusion did not materially influence the results. Nevertheless, the small size of the DD subgroup is acknowledged as a methodological limitation. Children with significant physical, visual, or auditory impairments that could hinder their ability to participate in the eye‐tracking tests were excluded to maintain the integrity of the data collection process.

### 2.2 Data Collection and Clinical Assessments

Data collected included sociodemographic information and detailed developmental and clinical histories. Families of participants were requested to share prior assessments, including information on IQ, language development, and other concerns. Screening for all participants was conducted using the Arabic and English versions of the Social Communication Questionnaire (SCQ) (Aldosari et al. 2019; Rutter 2003). Participants underwent further diagnostic evaluation using the Autism Diagnostic Observation Schedule, Second Edition (ADOS‐2) (Chandler et al. 2007; Hurwitz and Yirmiya 2014; Lord et al. 2012). The ADOS‐2 total, social effect, and restricted/repetitive behavior raw scores were converted into calibrated severity scores based on the specific module and comparison metrics for each participant.

These assessments were carried out by licensed members of the research team that include clinicians and psychologists who were formally trained and certified in ADOS‐2 administration. ADOS‐2 assessments were administered to 175 participants. ADOS‐2 was conducted only for children who had a prior clinical diagnosis of ASD or who screened positive on the SCQ or raised clinical concerns suggestive of ASD. TD participants without developmental or psychiatric concerns did not undergo ADOS assessment.

A consensus diagnosis was reached following a thorough review of medical history, psychosocial and developmental histories, and clinical assessments. Final diagnoses were established for all participants according to the Diagnostic and Statistical Manual of Mental Disorders, Fifth Edition (DSM‐5) criteria (Williams and First 2013) by experienced neurodevelopmental physicians specialized in ASD to ensure diagnostic accuracy and consistency across cases. Eye‐tracking acquisition was conducted independently of diagnostic evaluation. Staff administering the eye‐tracking paradigm were not involved in diagnostic decision‐making and were unaware of participants' diagnostic status at the time of testing. Diagnostic assessments were performed by licensed clinicians using standardized procedures and were not informed by eye‐tracking results.

### 2.3 Eye Tracking Stimuli Creation

The development of the short version of the eye‐tracking stimuli was driven by the need to accommodate attention limitations commonly observed in children with autism and to address caregiver feedback highlighting the challenges posed by the length of the original version. The initial version (Al‐Shaban et al. 2023), created in collaboration with Cleveland Clinic and adapted for cultural relevance, was reviewed to identify components most indicative of ASD. These key segments were carefully selected to form a more concise stimulus set, balancing diagnostic accuracy with practicality in implementation.

This revised version underwent the same rigorous process as the original longer version (Al‐Shaban et al. 2023). The updated stimulus paradigm was subsequently tested on a sample of children across diagnostic groups to evaluate whether it produced results consistent with those observed in the original version. By refining the length and focusing on the most diagnostic elements, the revised stimuli aim to enhance feasibility for both children and caregivers while preserving its clinical validity.

### 2.4 Computation of the Autism Index (AI)

**FIGURE 1:**
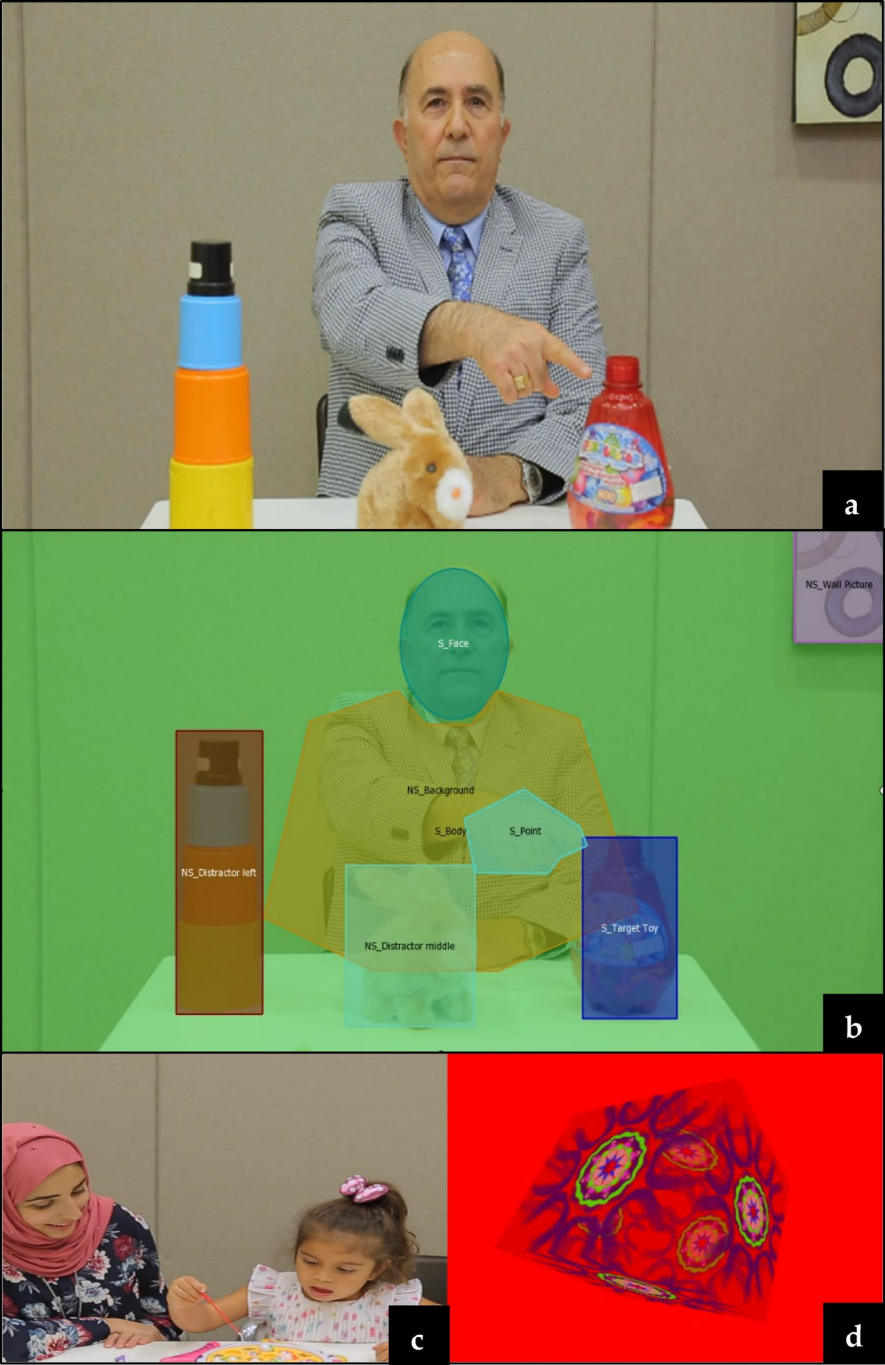
Example of stimuli used in the short eye‐tracking paradigm. (a) Example of a social video presented alone. (b) Illustration of regions of interest (ROIs) overlaid on the same stimulus, showing social (S) ROIs (e.g., face, body, pointing hand, and target object of joint attention) and non‐social (NS) ROIs (e.g., non‐referenced objects and background elements) as defined a priori for gaze classification. (c, d) Example of paired stimulus presentation, in which a social video (c) and a non‐social geometric motion video (d) were displayed simultaneously on the screen. Areas of interest (AOIs) were defined around the social and non‐social stimulus regions, and gaze allocation across these regions was used to compute the Autism Index.

The AI quantifies the ratio of gaze directed toward social versus non‐social regions across all valid stimuli, defined as stimulus presentations for which gaze data met minimum quality criteria (e.g., sufficient tracking ratio and fixation duration) to allow reliable computation of dwell proportions. Tracking ratio was calculated as the proportion of each stimulus presentation during which valid gaze samples were successfully recorded relative to the total stimulus duration. For each trial, the dwell proportion—the percentage of total fixation time within each ROI relative to total on‐screen viewing time—was computed after applying an 80 ms minimum fixation duration filter. Regions of interest (ROIs) were defined a priori based on stimulus content and categorized as social or non‐social. Social ROIs included faces, eyes, hands, bodies, and other socially relevant actions performed by actors within the scene. In stimuli depicting referential social cues, such as gaze direction or pointing gestures, the referenced target of joint attention (e.g., the object being indicated) was also classified as part of the social ROI, as successful social processing requires following the referential cue to its intended target. Non‐social ROIs included background elements, geometric patterns, or objects not referenced by a social cue. Figure 1 shows sample social and non‐social frames used in the final battery.

Individual stimulus scores were aggregated into a composite AI using a standardized algorithm validated in previous research (Frazier et al. 2018; Al‐Shaban et al. 2023). Higher AI values reflected stronger attention to non‐social content relative to social stimuli. Quality metrics such as overall tracking ratio and number of valid stimuli were recorded to assess data reliability.

Nineteen video clips were selected based on prior analyses of discriminative power and engagement. Each clip (5–10 s) depicted either dynamic social scenes (e.g., faces, joint attention, shared play) or non‐social motion (e.g., geometric or object‐based activity). Audio was intentionally excluded for most of the stimuli to minimize linguistic and cultural confounds. Depending on the trial, stimuli were presented either as a single video or as paired social and non‐social videos displayed simultaneously, allowing direct comparison of gaze allocation between stimulus types. Higher AI values reflected greater relative attention to non‐social content.

### 2.5 Eye Tracking Data Acquisition and Processing

Eye‐tracking data were collected in a quiet room using the SMI RED250 remote eye tracker system (SensoMotoric Instruments GmbH, Germany) which was attached to a 19 in. LCD monitor. The system captured binocular gaze, 3D eye position, pupil, and timestamp data at a sampling rate of 250 Hz. Calibration was performed automatically using 2‐, 5‐, or 9‐point methods, with position accuracy to 0.4°. The same calibration procedure was used for all participants, regardless of diagnostic group. Calibration acceptance criteria were identical across groups, and recordings that did not meet these criteria were repeated or excluded prior to data collection. Therefore, group differences in data quality metrics are unlikely to reflect calibration‐related differences in gaze accuracy. Our primary measurement was dwell proportion. However, we also recorded additional gaze metrics, including visits to regions of interest, average saccade length, peak saccade velocity, and blink frequency.

Following recommendations of Sasson and Elison (2012) (Sasson and Elison 2012), children were seated either alone or on their caregiver's lap at a distance of approximately 60–65 cm from the LCD monitor and viewed the stimuli subtending a visual angle of about 18.8°. The room had standard lighting and was sparse, with visual barriers to reduce distractions. After calibration, we instructed the children to pay attention to the video but to look wherever they wanted. Stimuli were presented using the SMI Experiment Center. Gaze data were recorded throughout a 4 min session that included initial and recurring calibration, along with multiple stimuli (Figure 1) covering a range of paradigms: dynamic individual faces, joint attention cues, gaze‐following, reciprocal interactions, dynamic social versus geometric visuals, and passive viewing of social‐object arrays. Indicators that showed less attention to social cues in children with ASD (which were given a negative value) were combined with indicators that showed more attention to non‐social cues in ASD‐affected individuals.

Quality control was applied at the stimulus level. Tracking ratio was calculated for each stimulus as the proportion of stimulus duration during which valid gaze samples were successfully recorded relative to the total stimulus length. A stimulus was considered valid if the participant demonstrated ≥ 40% on‐screen looking during that presentation. Stimuli below this threshold were excluded from analysis. AI scores were computed using valid stimuli only. Participants were included in the final analysis if they contributed 40% and above valid stimuli across the task (≥ 8 of 19 stimuli). Participants failing to meet this criterion were excluded (*n* = 3).

Test–retest reliability was assessed for 19 participants across all groups (See Table S1), with an average interval of 2.4 months between tests, demonstrating the consistency of the eye‐tracking measures over time.

### 2.6 Statistical Analysis

All analyses were conducted using IBM SPSS Statistics v29. Outliers and high‐leverage cases were identified through examination of univariate and bivariate distributions. Analyses were run both with and without these cases; as the results did not differ meaningfully, all data were retained for analysis. Descriptive statistics were used to summarize sample characteristics. Due to the automated nature of eye‐tracking acquisition and AI computation, blinding was not required; diagnostic assessments were conducted independently of eye‐tracking results. To compare baseline and retest participants, Chi‐square tests were applied for categorical variables and independent samples *t*‐tests were used for continuous variables. Cohen's d was reported to reflect the effect size of group differences. Potential floor effects due to low overall screen engagement were mitigated by computing the AI only from valid stimuli that met predefined eye‐tracking quality thresholds, and by using dwell proportions rather than absolute viewing time. Participants with insufficient tracking were excluded at the stimulus level, and sensitivity analyses confirmed that results were robust to stricter tracking ratio thresholds.

The validity of the AI tool, SCQ, and ADOS‐2 severity scores was assessed using receiver operating characteristic (ROC) curve analysis. The area under the ROC curve (AUC) served as the primary indicator of diagnostic accuracy, with a 95% confidence interval above 0.80 considered evidence of strong validity. Concurrent validity was assessed using Spearman's rank‐order correlations among the AI, SCQ, and ADOS‐2 scores. Diagnostic accuracy was evaluated using a case–control design including children with ASD and non‐ASD controls, allowing estimation of sensitivity, specificity, and overall discriminative performance. No fixed diagnostic cutoff was prespecified for the AI; diagnostic performance was primarily evaluated using AUC to avoid reliance on a single threshold. To account for developmental variability, diagnostic performance was examined using age‐stratified analyses. Participants or stimulus presentations with insufficient eye‐tracking data were excluded as predefined in the quality control criteria.

Internal consistency reliability of the AI and its attention indices was estimated using Cronbach's alpha, and test–retest reliability was evaluated using Pearson's *r*. For the validation of the AI, Spearman correlations were used to examine associations between social and non‐social attention indices and the SCQ raw scores, ADOS‐2 total scores, and calibrated severity scores (calculated based on established norms).

Because the long and short versions were evaluated in different cohorts, direct statistical comparison between versions was not feasible; accordingly, comparisons are descriptive and based on overall performance metrics. All statistical tests were conducted using a Type I error rate of 0.05. To control for multiple comparisons, the Benjamini–Hochberg false discovery rate correction was applied. Additionally, only correlations greater than *r* = 0.40 were considered clinically significant. The validation sample was deliberately overpowered to detect a meaningful AUC exceeding 0.80.

## 3 Results

### 3.1 Sample Description

The final baseline sample consisted of 236 participants (127 ASD, 109 non‐autistic), while the retest sample included 19 participants (13 ASD, 6 non‐autistic). Table 1 summarizes participant characteristics and eye‐tracking data quality for the baseline ASD and control groups. The ASD and control groups were comparable in age, but differed in clinical severity, with higher SCQ total raw scores and ADOS‐2 severity scores observed in the ASD group (both *p* < 0.001). With respect to eye‐tracking feasibility, children with ASD demonstrated lower overall tracking ratios (77.0% ± 16 vs. 90.2% ± 7.7%) and a lower proportion of valid stimuli compared (84.2% ± 12.5% vs. 94.7% ± 10.0%) with controls (both *p* < 0.001), indicating reduced data yield during testing but sufficient signal quality for analysis. Effect size estimates indicated large group differences for eye‐tracking quality metrics, supporting their relevance for group‐level differentiation.

**TABLE 1:** Participant characteristics and eye‐tracking quality in ASD and control groups.

	ASD	Controls	
	*M* (SD)	*M* (SD)	*t*/*χ* ^2^ (*p*)
*N*	127	109	
Age	6.68 (3.37)	6.10 (3.34)	−1.33 (0.185)
Male (*N*, %)	105 (82.7%)	62 (56.9%)	18.87 (< 0.001)
Comorbidities (*n*, %)			
Verbal delay	62 (48.8%)	17 (15.2%)	28.94 (< 0.001)
GDD/ID	75 (59.1%)	16 (14.3%)	48.71 (< 0.001)
Anxiety disorder	41 (32.3%)	22 (19.6%)	4.27 (0.039)
ADHD	46 (36.2%)	15 (13.4%)	15.14 (< 0.001)
Other	34 (26.8%)	15 (13.4%)	5.74 (0.017)
SCQ total raw score	18.46 (7.09)	6.83 (6.16)	−8.27 (< 0.001)
ADOS	6.18 (1.6)	1.67 (1.5)	−19.03 (< 0.001)
Overall tracking ratio (%)	77.03% (15.96)	90.21% (7.72)	7.21 (< 0.001)
Number of valid stimuli	84.2% (12.5%)	94.7% (10.0%)	6.47 (< 0.001)

Age, gender distribution, SCQ total raw scores, and ADOS‐2 severity scores were comparable across baseline and retest samples, with no statistically significant differences observed (See Table S2). However, the retest group showed a significantly higher proportion of individuals with verbal delay (66.7% vs. 33.1%, *p* = 0.002). Overall, both the baseline and retest samples included participants with diverse ages and levels of severity, presenting a relatively complex and heterogeneous population for diagnostic differentiation.

### 3.2 Autism Index Score Range and Reliability

**FIGURE 2:**
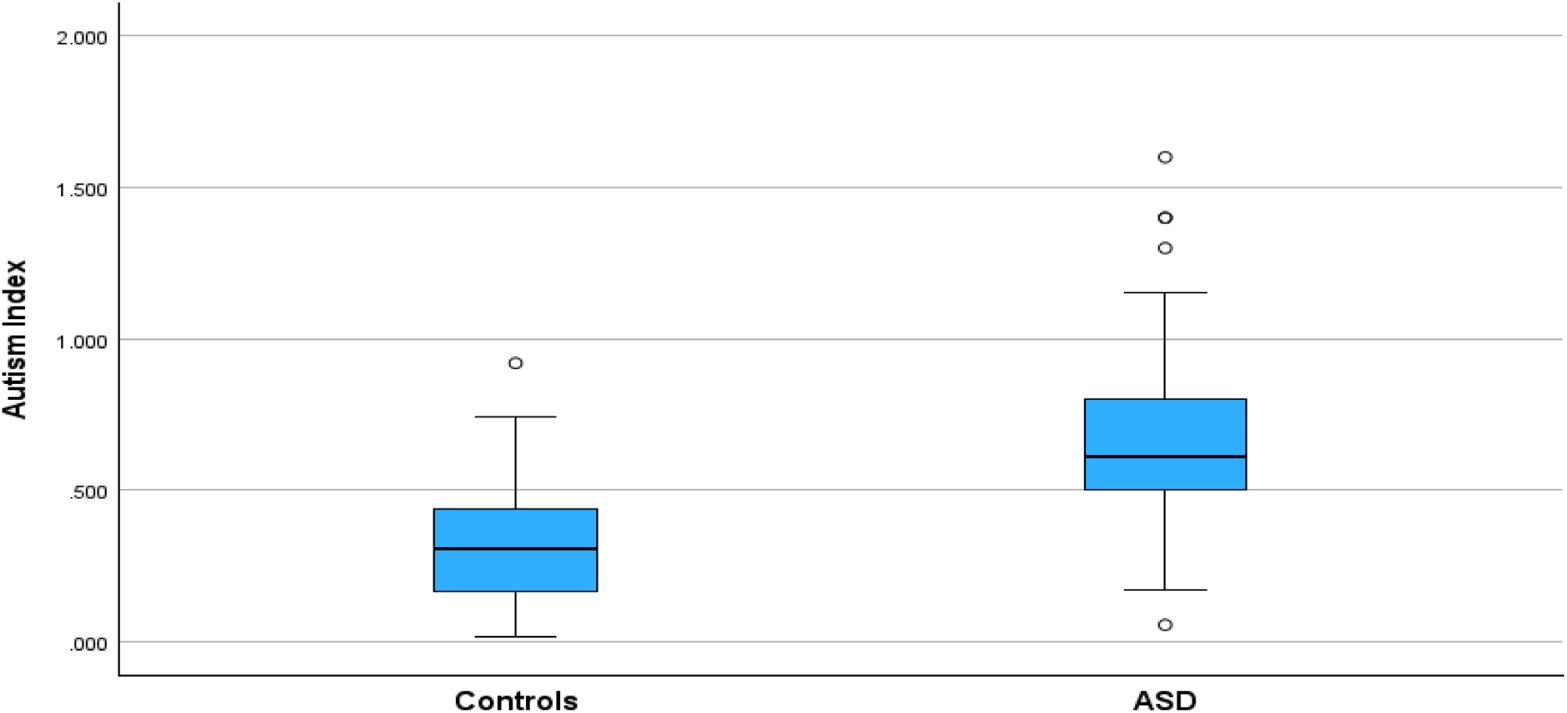
Boxplot of Autism Index scores for ASD diagnosed and non‐autistic controls.

**FIGURE 3:**
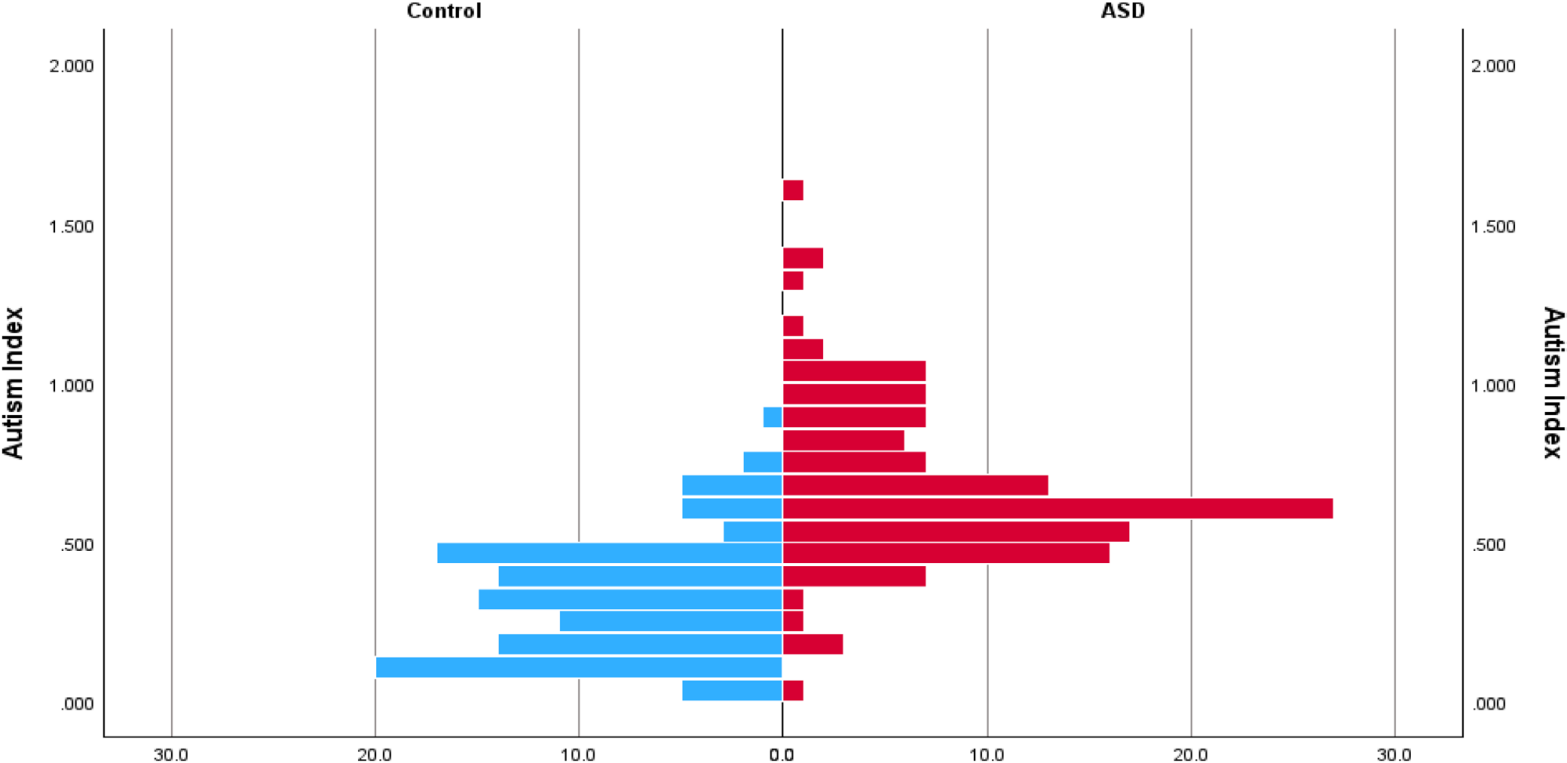
Side by side histogram of Autism Index scores or less compliant ASD cases.

AI scores were moderately skewed in the ASD group (skew = 0.87, kurtosis = 1.71) and mildly skewed in the control group (skew = 0.58, kurtosis = −0.07). Participants demonstrated a wide range of scores on the AI (range: 0.019 to 1.600), with a significant upward shift in the ASD group (Cohen's d = −1.55, *p* < 0.001), indicating a large effect size. There was a statistically significant difference in AI scores between ASD and control participants, with ASD individuals showing substantially higher scores (Figures 2 and 3).

When considered separately, the social and non‐social attention indicators had very good to excellent internal consistency and test–retest reliability (social attention: *α* = 0.90, *r* = 0.73; non‐social attention: *α* = 0.80, *r* = 0.44). The AI showed strong test–retest reliability over a 1–2 month interval (*α* = 0.91, *r* = 0.83, *p* < 0.001), indicating consistent performance across repeated administrations (Table 2).

**TABLE 2:** Internal consistency and test–retest reliability coefficients for social and non‐social attention indicators and the autism index.

	*k* (Number of indicators)	Internal consistency *α*	Test–retest *r*
Social attention	177	0.9	0.73
Non‐social attention	195	0.8	0.44
Autism index	372	0.91	0.834

### 3.3 Diagnostic and Concurrent Validity

**FIGURE 4:**
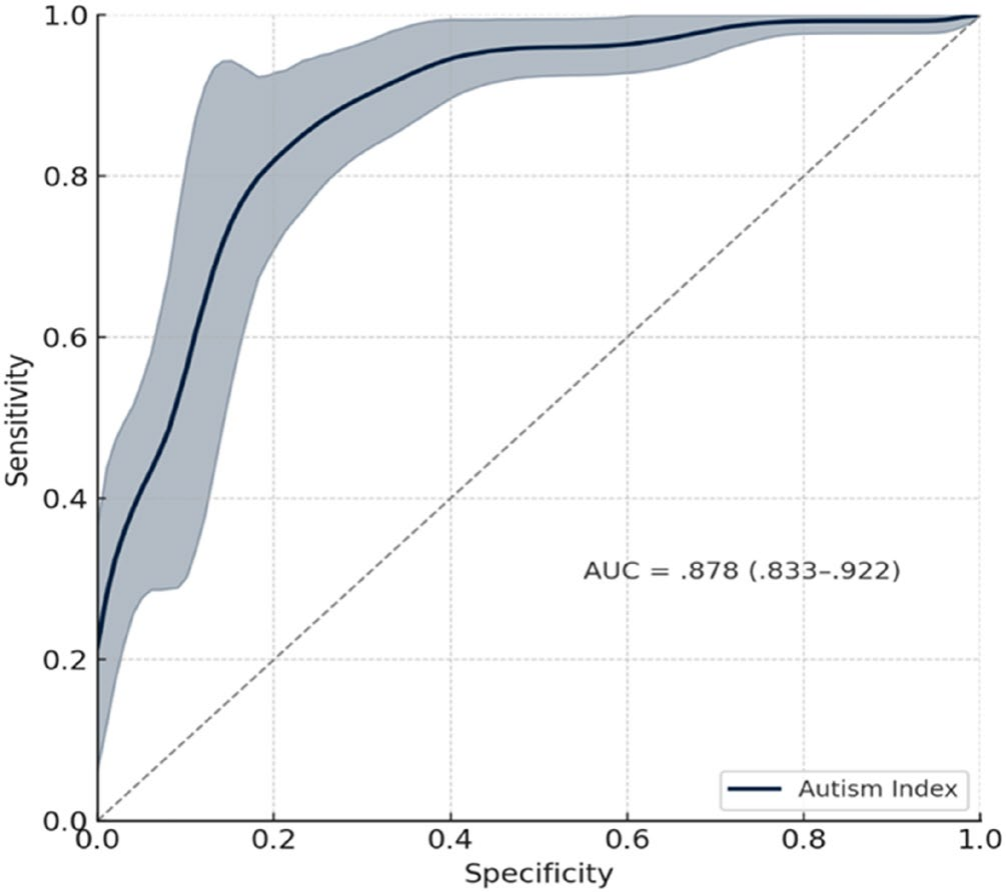
Receiver operating characteristics curve for the Autism Index predicting clinical ASD diagnosis.

AI scores demonstrated excellent diagnostic accuracy in distinguishing ASD from controls, as reflected by a high area under the curve (AUC = 0.878, SE = 0.023) (Figure 4). The ROC curve showed strong sensitivity and specific performance across a range of cut‐offs, indicating the robustness of the measure. Evaluating a stricter subset by excluding participants with a tracking ratio < 80% (*n* = 67) yielded a slightly higher diagnostic accuracy (AUC = 0.887 vs. 0.878). However, this minimal improvement suggests that the overall model performance remained stable, while such exclusion may still limit generalizability by removing more affected.

ROC performance was further analyzed across age groups (Table 3). In the ≤ 3‐year group, AUC was 0.932 (SE 0.042). In the > 3–6‐year group, AUC was 0.880 (SE 0.034). In the > 6–10‐year group, AUC was 0.939 (SE 0.026). In the > 10‐year group, AUC was 0.846 (SE 0.067).

**TABLE 3:** Age‐stratified AUC for the Autism Index.

Age in years	ASD	Controls	Total	AUC	SE	95% CI	*p*
≤ 3	14	24	38	0.932	0.042	0.849–1.000	< 0.001
> 3–6	50	43	93	0.880	0.034	0.813–0.947	< 0.001
> 6–10	42	30	72	0.939	0.026	0.889–0.990	< 0.001
> 10	21	15	36	0.846	0.067	0.714–0.978	< 0.001

**FIGURE 5:**
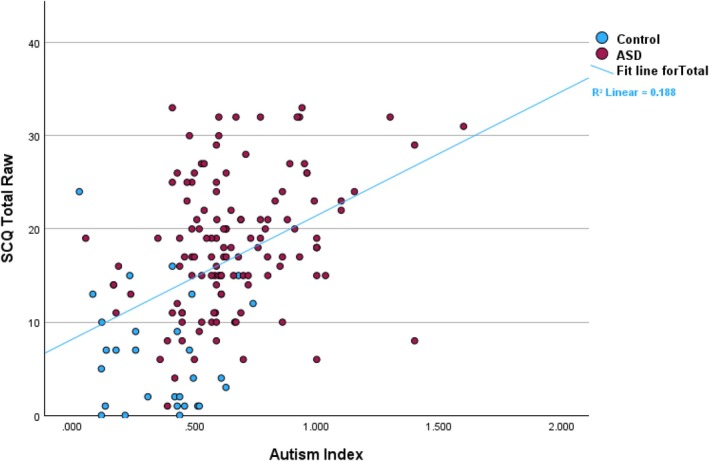
Bivariate correlation between the Autism Index and SCQ total raw scores.

**FIGURE 6:**
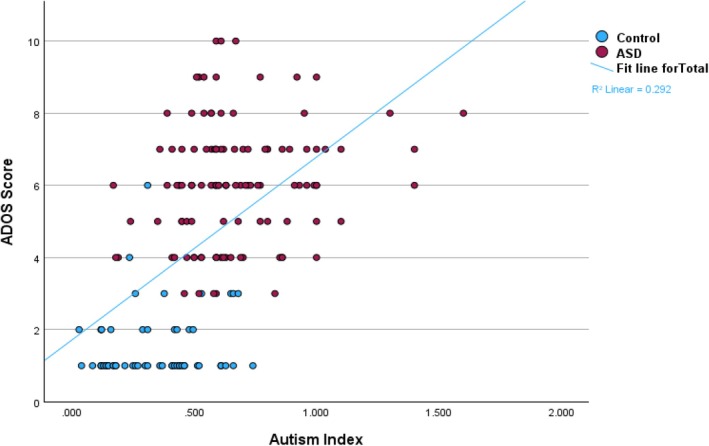
Bivariate correlation between the Autism Index and ADOS scores.

To assess consistency across assessment measures, AI scores were examined in relation to both clinician‐rated (ADOS‐2) and parent‐reported (SCQ) symptom severity. AI scores were significantly positively correlated with SCQ total raw scores in the full sample (*r* = 0.43, *p* < 0.001) (Figure 5). Moreover, AI scores showed a significant positive correlation with ADOS‐2 severity scores (*r* = 0.54, *p* < 0.001) (Figure 6) among the 175 participants with available ADOS data. This supports the concurrent validity of the AI as an indicator of autism‐related symptom severity.

### 3.4 Concordance Between Eye‐Tracking Results and Clinical Diagnosis in Referred ASD Cases

This subsection examines concordance between eye‐tracking outcomes and established clinical diagnoses in a clinically referred sample. Diagnostic accuracy estimates (e.g., AUC) are reported for the full case–control cohort in Sections 3.1, 3.3. To further evaluate the clinical validity of the short version eye‐tracking tool, a subset of 42 participants referred from specialized centers underwent evaluation using the ADOS‐2, and their results were compared with the eye‐tracking outcomes (See Table S3). These referrals originated from top medical institutions in Qatar such as Hamad Medical Corporation (HMC), The Child Development Center (CDC), Sidra Medicine, and other specialized centers, where participants had previously received a confirmed ASD diagnosis. The ADOS‐2 modules administered ranged from Module 1 to Module 4, covering a wide spectrum of language and developmental levels. Severity scores spanned from 2 (minimal‐to‐no evidence) to 10 (high), with the majority of participants falling within the moderate‐to‐high severity range.

Of the 42 participants, 40 had an established ASD diagnosis prior to enrollment. Most cases showed strong alignment between prior diagnosis, ADOS‐2 severity classification, and a positive eye‐tracking result, reinforcing the concurrent validity of the short version paradigm. Only three cases showed a mismatch between clinical diagnosis and eye‐tracking result, all of which were classified as “false negative.” This corresponds to a false negative rate of 7.1%. These outliers may reflect individual variability, low symptom severity, or reduced attentional engagement during the brief eye‐tracking session.

## 4 Discussion

This study evaluated the reliability and diagnostic validity of a 4 min eye‐tracking paradigm designed to identify (ASD Building on our previously validated long‐version paradigm (Al‐Shaban et al. 2023)), the present version demonstrates that comparable diagnostic performance can be achieved using a substantially shorter and more feasible assessment. The results demonstrated that the short version of the AI was capable of effectively distinguishing between diagnostic groups, supporting its potential utility as a screening tool and diagnostic assessment. Unlike earlier versions of this AI, which were developed based on non‐Arabic populations and stimuli, the current version represents a significant advancement—designed for Arabic and English‐speaking children using culturally relevant stimuli. This adaptation addresses a key limitation of prior models, which lacked linguistic and contextual specificity, thereby enhancing ecological validity and supporting diagnostic performance within Arabic and English‐speaking populations (Al‐Shaban et al. 2023).

The findings of the current study suggest that AI may serve as a valuable tool when used alongside commonly employed clinical measures for identifying ASD (Bishop and Seltzer 2012). This suggests that the eye tracking technology may capture complementary features of ASD that reflect underlying attentional processes not directly assessed by traditional behavioral measures (Pierce et al. 2016). Unlike conventional tools such as the ADOS or SCQ, which depend on clinician interpretation or parent‐reported observations (Chevallier et al. 2015), the AI captures implicit attentional mechanisms underlying social motivation and perceptual processing. The significant correlation between AI scores and standardized diagnostic measures, coupled with its strong discriminative accuracy, supports its potential role as a complementary biomarker that provides an additional, biologically grounded dimension of ASD assessment. The strong correlation between the AI and SCQ scores shows that the AI differentiates symptom severity in a way that aligns with established screening measures, supporting its validity (Aldosari et al. 2019; Berument et al. 1999; Corsello et al. 2007). Moreover, age‐stratified AUCs (≤ 3, > 3–6, > 6–10, > 10 years) ranged from 0.846 to 0.939 (SE 0.026–0.067), demonstrating stable discriminative performance across developmental stages. These findings support the tool's potential applicability across a wide age range.

The AI reflects how visual attention is distributed between social and non‐social dynamic content, capturing implicit attentional patterns related to social interest and perceptual prioritization. Reduced spontaneous attention to social information is a well‐documented feature of ASD and has been linked to differences in social motivation, attentional orienting, and processing of socially salient cues (Chita‐Tegmark 2016; Kaliukhovich et al. 2020). Studies have shown that reduced attention to faces and eyes not only differentiates autistic from non‐autistic children but also predicts later social symptom severity, supporting the interpretation of gaze allocation as a core, trait‐like feature of ASD rather than a task‐specific effect (Jones et al. 2008; Jones and Klin 2013). Although the current paradigm contrasts social and non‐social stimuli, it does not isolate specific perceptual mechanisms, and differences in low‐level visual features between stimulus categories represent an important limitation that future stimulus‐matched designs should address. Consistent with these attentional characteristics, the observed differences in data quality between groups are expected (i.e., tracking ratio), as children with ASD often exhibit reduced attention, greater movement, and variable gaze behavior during testing as compared to neurotypical individuals (Chita‐Tegmark 2016; Kaliukhovich et al. 2020). Such variability reflects inherent features of the condition rather than technical limitations of the device. Importantly, despite these differences, the model maintained high diagnostic accuracy, indicating that the eye‐tracking algorithm effectively distinguishes ASD‐related gaze patterns even under less optimal recording conditions. The high internal consistency and reproducibility across sessions further support the reliability and potential clinical relevance of the short version AI for identifying ASD‐related attentional patterns. Furthermore, the observed test–retest reliability provides preliminary support for the temporal stability of the measure, although confirmation in larger retest samples is warranted. While previous studies on gaze‐based metrics have shown mixed findings (Chawarska et al. 2013), with most reporting only moderate reliability (Farzin et al. 2011), the current findings indicate a higher degree of reliability. This suggests that AI captures stable, trait‐like attentional behaviors that align with core features of ASD.

Our findings are consistent with recent work by Jones and Klin (2013), who demonstrated that eye‐tracking–based measures of social visual engagement can provide clinically meaningful diagnostic information during early development, including in children aged 18–30 months (Jones, Klaiman, Richardson, Aoki, et al. 2023; Jones, Klaiman, Richardson, Lambha, et al. 2023). In those studies, social attention metrics showed strong agreement with expert clinical diagnosis and robust diagnostic accuracy in early childhood populations. Importantly, they also reported that eye‐tracking–derived social attention scores varied systematically with both social and cognitive developmental level, indicating sensitivity to broader developmental trajectories rather than categorical diagnosis alone. The present findings extend this body of work by demonstrating comparable discriminative performance using a substantially shorter paradigm that is culturally adapted for Arabic‐ and English‐speaking populations and spans a wider age range. Together, these results support the growing evidence base for eye‐tracking as a scalable, objective complement to traditional clinical diagnostic assessments.

In addition, the present findings closely align with the brief social attention paradigm reported by Frazier et al., who demonstrated that a 4 min eye‐tracking measure can reliably capture social attention patterns across neurogenetic syndromes and idiopathic neurodevelopmental disability using remote assessment (Frazier et al. 2025). Importantly, both studies independently converge on the conclusion that very rapid eye‐tracking paradigms are sufficient to yield reliable objective measures of social attention. However, the two approaches differ in important aspects. Whereas Frazier et al. emphasized transdiagnostic characterization and remote monitoring across heterogeneous neurodevelopmental populations, the current study focused specifically on ASD detection and concurrent clinical validity using gold‐standard diagnostic instruments (ADOS‐2 and SCQ) within a clinic‐based setting. Together, these complementary findings strengthen the evidence that brief eye‐tracking paradigms can serve both as scalable screening tools and as clinically grounded adjuncts to formal ASD diagnosis.

Among participants referred from ASD specialized centers, the eye‐tracking tool exhibited a false negative rate of 7.1%, indicating that while it successfully identified most clinically confirmed ASD cases, a small subset was incorrectly classified. These misclassifications highlight the importance of investigating underlying factors—such as fluctuations in attentional engagement or individual variability in visual processing—that may compromise diagnostic accuracy in certain cases. Reduced and inconsistent social visual attention has been well documented in children with ASD, characterized by slower orientation to faces and decreased dwell time on social stimuli (Hochhauser et al. 2021; McLaughlin et al. 2021). Moreover, variability in gaze patterns—such as inconsistent fixation on diagnostic regions like the eyes—has been associated with heterogeneity in symptom presentation and may impact the sensitivity of the eye‐tracking paradigms in some cases (Cilia et al. 2021; McPartland et al. 2011). Nevertheless, this subgroup analysis reinforces the strong concordance between standardized diagnostic evaluations, detailed clinical histories, and objective gaze‐based measures, while also emphasizing the need for continued refinement of the tool to enhance sensitivity in cases with subthreshold symptoms or reduced task engagement.

Notably, the current model version demonstrated a higher diagnostic accuracy compared to the previous version (Al‐Shaban et al. 2023) (Table 4), while utilizing a shorter and more efficient testing paradigm. Around 89% of participants produced valid stimuli data, and none required test termination due to fatigue or disengagement. This suggests that the 4 min format accommodates the attentional limitations commonly seen in children with ASD and increases the practicality of the tool for routine clinical or screening use. It also shows strong convergent validity through significant correlations with established clinical measures, including ADOS‐2 and SCQ scores. Comparisons with the original paradigm are necessarily descriptive, as the two versions were evaluated in independent cohorts; nevertheless, the observed performance metrics indicate that comparable and potentially enhanced diagnostic performance can be achieved with the short paradigm.

**TABLE 4:** Comparison between the long and short versions of the eye‐tracking paradigm.

Aspect	Long version (Al‐Shaban et al. 2023)	Short version (current study)
Stimuli	44	19
Test duration (min)	~10 min	~4 min
Sample size	240 participants (ASD = 144, Controls = 96 [NA + DD])	236 participants (ASD = 127, Controls = 109 [TD + DD])
AUC	0.730 (SE = 0.035)	0.878 (SE = 0.023)
Test–retest reliability	*r* = 0.73	*r* = 0.83
ADOS–AI correlation	*r* = 0.10 (ns)	*r* = 0.54 (*p* < 0.001)
SCQ–AI correlation	*r* = 0.46 (*p* < 0.001)	*r* = 0.43 (*p* < 0.001)
Feasibility	Mean valid stimuli = 35/44 stimuli, ≈80%	Mean valid stimuli = 17/19 stimuli, ≈89%

Our findings underscore the potential of short, objective eye‐tracking assessments as scalable tools for early ASD screening and adjunctive diagnostic assessment, particularly in resource‐limited or culturally diverse settings where access to trained clinicians is restricted. A 4 min paradigm may be suitable for integration into pediatric visits, early‐intervention centres, or community‐based screening programs, pending further validation. Additionally, because the paradigm generates standardized quantitative metrics, it may complement behavioral assessments in longitudinal monitoring, intervention trials, and cross‐cultural research. The current results further support the feasibility of implementing eye‐tracking protocols in Arabic‐speaking populations, contributing to global equity in ASD diagnostics.

## 5 Limitations

Although the findings are promising, the current study is not without limitations. The paradigm was administered to children who were able to successfully complete the task, potentially underrepresenting individuals with more pronounced behavioral or attentional challenges. In addition, although the majority of control participants were neither siblings of autistic individuals nor identified as high‐risk, the inclusion of some siblings remains a methodological limitation. Moreover, given that most controls were TD, estimates likely represent upper‐bound performance pending validation in more diverse samples. Social and non‐social stimuli differed in low‐level visual properties (e.g., color and contrast), which may contribute to attentional differences independent of social content. Group differences in eye‐tracking data quality metrics were observed, which may reflect characteristic differences in engagement and gaze behavior across diagnostic groups. Detailed descriptive statistics for raw dwell time and total screen time were not retained for all participants in the current dataset, limiting retrospective reporting of these measures. Future studies will prioritize comprehensive archiving of stimulus‐level gaze metrics to facilitate detailed descriptive and comparative analyses. In addition, the test–retest analysis was based on a small subset of participants and should be considered exploratory. Finally, the broad age range, while intentionally included to assess robustness across development, may introduce developmental heterogeneity that warrants further age‐specific validation. Future research should seek to replicate these findings in larger cohorts to enhance generalizability and robustness of the results.

## 6 Conclusion

The current study provides compelling evidence that a 4 min eye‐tracking paradigm can maintain—and even enhance—the diagnostic accuracy of longer tests while significantly improving feasibility and user experience. The short version was developed to reduce testing duration and cognitive demand, as individuals with autism often struggle to maintain attention during lengthy assessments. This improvement enhances the feasibility and usability in clinical and community‐based settings. Moreover, it demonstrated strong reliability, robust correlations with established clinical measures, and solid diagnostic accuracy. It offers a scalable and objective approach to ASD screening and diagnosis in multicultural populations.

Future efforts should focus on integrating the tool into real‐world clinical workflows, validating its performance across broader and more diverse populations, and developing implementation guidelines to support consistent interpretation and use. With continued refinement and expansion, this tool has the potential to become a key component in advancing early and accessible ASD detection.

## Funding

This research was funded by Qatar Biomedical Research Institute under Grant No. (SP001172).

## Ethics Statement

The study was conducted in accordance with the Declaration of Helsinki and approved by the Institutional Review Board of Hamad Bin Khalifa University (HBKU). Written informed consent was obtained from all participants' parents or legal guardians prior to participation in the study.

## Consent

The authors have nothing to report.

## Conflicts of Interest

The authors declare no conflicts of interest.

## Supporting information


**Table S1:** Details of period between test–retest samples.
**Table S2:**. Participant characteristics in the baseline and retest sample.
**Table S3:** ADOS‐2 severity and eye‐tracking results in referred ASD cases.

## Data Availability

The data that support the findings of this study are available on request from the corresponding author. The data are not publicly available due to privacy or ethical restrictions.
