# Supplementary material for: Development and Validation of a Short Version Eye‐Tracking Paradigm for the Screening and Diagnosis of Autism Spectrum Disorder in Qatar
Source: Autism Res. 2026 Mar 30;19(6):e70242. doi: 10.1002/aur.70242 (PMC13276692; doi:10.1002/aur.70242)
Supplement: Supplementary file 1 — Table S1: Details of period between test–retest samples. Table S2:. Participant characteristics in the baseline and retest sample. Table S3: ADOS‐2 severity and eye‐tracking results in referred ASD cases. [file AUR-19-0-s001.docx]

**Supplementary Materials**

**Table S1**. Details of period between test-retest samples.

| **Test** | **Retest** | **Number of days** | **Months** |
| --- | --- | --- | --- |
| 02-11-23 | 03-12-23 | 29 | 1 |
| 12-13-23 | 11-01-24 | 324 | 10.8 |
| 01-07-24 | 1-21-2024 | 14 | 0.5 |
| 01-07-24 | 1-21-2024 | 14 | 0.5 |
| 01-10-24 | 1-16-2024 | 6 | 0.2 |
| 01-28-2024 | 2-15-2024 | 18 | 0.6 |
| 01-29-2024 | 2-14-2024 | 16 | 0.5 |
| 02-06-24 | 2-21-2024 | 15 | 0.5 |
| 03-06-24 | 3-27-2024 | 21 | 0.7 |
| 06-02-23 | 5-13-2024 | 346 | 11.5 |
| 04-30-2024 | 05-09-24 | 9 | 0.3 |
| 08-11-24 | 8-15-2024 | 4 | 0.1 |
| 08-11-24 | 8-15-2024 | 4 | 0.1 |
| 01-05-25 | 01-06-25 | 1 | 0 |
| 07-16-2023 | 5-16-2024 | 305 | 10.2 |
| 01-09-24 | 6-25-2024 | 168 | 5.6 |
| 1-15-2024 | 02-12-24 | 28 | 0.9 |
| 06-10-24 | 6-25-2024 | 15 | 0.5 |

**Table S2**. Participant characteristics in the baseline and retest sample.

|  | **Baseline** | **Retest** | **χ²/t (p-value)** | **Cohen’s d** |
| --- | --- | --- | --- | --- |
| **Total N** | 236 | 19 | 2.66 (0.103) | 0.206 |
| **Non-Autistic (n,%)** | 109 (46.2%) | 6 (31.6%) |  |  |
| **Autism Spectrum Disorder (n, %)** | 127 (53.8%) | 13(68.42%) |  |  |
| **Age (SD, range)** | 6.4 (±3.35, 1.3–16.5) | 4.9 (±3, 2.4-14.6) | 1.86 (.064) | 0.49 |
| **Gender (n, %)** |  |  |  |  |
| **Female** | 69 (29.2%) | 2 (11.1%) | 3.1 (0.079) | 0.223 |
| **Male** | 167 (70.8%) | 16 (88.9%) |  |  |
| **ASD ratio (female: male)** | 1: 4.8 | 1:12 | 0.36 (.550) | 0.102 |
| **Comorbidities (n, %)** |  |  |  |  |
| **Verbal Delay** | 78 (33.1%) | 12 (66.7%) | 9.95 (.002) | 0.412 |
| **GDD/ID** | 90 (38.1%) | 11 (61.1%) | 4.36 (.037) | 0.226 |
| **Anxiety Disorder** | 63 (26.7%) | 4 (22.2%) | 0.20 (.655) | 0.056 |
| **ADHD** | 61 (25.8%) | 4 (22.2%) | 0.13 (.715) | 0.046 |
| **Other** | 49 (20.8%) | 4 (22.2%) | 0.03 (.874) | 0.022 |
| **SCQ total raw score** |  |  |  |  |
| **Non-autistic** | 6.8 (6.15) | 4.67 (4.72) | 0.86 (.361) | –0.37 |
| **Autism spectrum disorder** | 18.5 (7.1) | 19 (3.8) |  |  |
| **ADOS-2 Total Severity** | 6.0 (1.7) | 6.18 (1.6) | –0.288 (.774) | –0.09 |
| **Overall Tracking Ratio (%)** | 82.4% (14.7%) | 79.88% (16.5%) | –0.28 (0.781) | 0.18 |
| **Number of Valid Stimuli**  **(out of 19)** | 17.0 (3.2) | 16.5 (3.7) | 0.57 (.574) | 0.15 |

**Table S3.** ADOS-2 Severity and Eye-Tracking Results in Referred ASD Cases

| **Participant** | **ADOS-2 Module** | **ADOS-2 Severity Score** | **Confirmed ASD Diagnosis** | **Referring Institution** | **Eye tracking Result** | **Medical Record Available** |
| --- | --- | --- | --- | --- | --- | --- |
| **1** | 1 | 6-MODERATE | YES | CDC | POSITIVE | YES |
| **2** | 2 | 7-MODERATE | YES | HMC | POSITIVE | YES |
| **3** | 1 | 9-HIGH | YES | NEDAA CENTER | POSITIVE | YES |
| **4** | 2 | 6-MODERATE | YES | HMC | POSITIVE | YES |
| **5** | 1 | 6-MODERATE | YES | HMC | POSITIVE | YES |
| **6** | 1 | 9-HIGH | YES | HMC | POSITIVE | YES |
| **7** | 1 | 7-MODERATE | YES | HMC | POSITIVE | YES |
| **8** | 1 | 9-HIGH | YES | SIDRA | POSITIVE | YES |
| **9** | 1 | MODERATE-TO-HIGH | YES | INDIA | POSITIVE | YES |
| **10** | 4 | 3-LOW | NO | HMC | FALSE NEGATIVE | YES |
| **11** | 2 | 6-MODERATE | YES | HMC | FALSE NEGATIVE | YES |
| **12** | 1 | 7-MODERATE | YES | HMC + CDC | POSITIVE | YES |
| **13** | 1 | 3-LOW | YES | AL AHLI | POSITIVE | YES |
| **14** | 1 | 7-MODERATE | YES | HMC | POSITIVE | YES |
| **15** | 1 | 6-MODERATE | YES | HMC | POSITIVE | YES |
| **16** | 1 | 10-HIGH | YES | HMC + CDC | POSITIVE | YES |
| **17** | 1 | 10-HIGH | YES | CDC | POSITIVE | YES |
| **18** | 1 | 6-MODERATE | YES | HMC | POSTIVIE | YES |
| **19** | 2 | 4-LOW | YES | CDC | POSITIVE | YES |
| **20** | T | 7-MODERATE | YES | SIDRA | POSITIVE | YES |
| **21** | 3 | 7-MODERATE | YES | HMC | POSITIVE | YES |
| **22** | 1 | 9-HIGH | YES | HMC | POSITIVE | YES |
| **23** | 1 | 7-MODERATE | YES | HMC | POSITIVE | YES |
| **24** | 1 | 3-LOW | YES | HMC | POSITIVE | YES |
| **25** | 2 | 7-MODERATE | YES | QISH | POSITIVE | YES |
| **26** | 1 | 7-MODERATE | YES | HMC | POSITIVE | YES |
| **27** | 1 | 7-MODERATE | YES | HMC | POSITIVE | YES |
| **28** | 1 | 10-HIGH | YES | HMC | POSITIVE | YES |
| **29** | 1 | 5-MODERATE | YES | HMC | POSITIVE | YES |
| **30** | 1 | 7-MODERATE | YES | HMC | POSITIVE | YES |
| **31** | 1 | 8-HIGH | YES | HMC | POSITIVE | YES |
| **32** | 3 | 6-MODERATE | YES | SIDRA + SHAFALLAH | POSITIVE | YES |
| **33** | 3 | 3-LOW | YES | HMC | FALSE NEGATIVE | YES |
| **34** | 1 | 6-MODERATE | YES | CDC | POSITIVE | YES |
| **35** | 1 | 8-HIGH | YES | CDC | POSITIVE | YES |
| **36** | 1 | 9-HIGH | YES | HMC | POSITIVE | YES |
| **37** | 1 | 7-MODERATE | YES | HMC | POSITIVE | Yes |
| **38** | 1 | 7-MODERATE | YES | HMC | POSITIVE | YES |
| **39** | 2 | 2-MINIMAL-TO-NO EVIDENCE | NO | CDC | NEGATIVE | Yes |
| **40** | 1 | 6-MODERATE | YES | HMC | POSITIVE | YES |
| **41** | 2 | 6-MODERATE | YES | HMC | POSITIVE | YES |
| **42** | 2 | 3-LOW | YES | SIDRA | POSITIVE | YES |
